# Unilateral Optic Neuritis: A Rare Complication after Measles-Mumps-Rubella Vaccination in a 30-Year-Old Woman

**DOI:** 10.1155/2016/8740264

**Published:** 2016-04-19

**Authors:** Chiara De Giacinto, Elvira Guaglione, Pia E. Leon, Rossella D'Aloisio, Odilla Vattovani, Giuseppe Ravalico, Daniele Tognetto

**Affiliations:** University Eye Clinic of Trieste, Ospedale Maggiore, Piazza dell'Ospitale 1, 34125 Trieste, Italy

## Abstract

*Purpose*. To report a case of unilateral optic neuritis following Measles-Mumps-Rubella (MMR) vaccination.* Methods*. A 30-year-old female developed unilateral optic neuritis five days after a Measles-Mumps-Rubella (MMR) booster vaccination. The patient displayed unilateral involvement, with severe visual loss. However, visual acuity improved significantly after four days of intravenous steroid therapy with 500 mg/day of methylprednisolone.* Conclusions*. Optic neuritis is one of the rare complications associated with the mumps, measles, and rubella vaccine. It may be a toxic reaction to the nonviral component of the vaccine, but the exact etiology is unknown. Postvaccination neuritis is generally bilateral and usually affects children. In adults, unilateral optic neuritis is usually correlated with multiple sclerosis (MS).

## 1. Introduction

Optic neuritis is an inflammatory, infectious, or demyelinating disorder affecting the optic nerve. Bilateral optic neuritis usually affects children and often follows viral infections, while it rarely affects adult patients not suffering from systemic inflammatory or autoimmune disease. In adults, optic neuritis is typically unilateral and is correlated with multiple sclerosis (MS) [1].

In rare cases optic neuritis may develop after vaccination injections. Postvaccination neuritis is generally bilateral and only rarely unilateral. The medical literature includes cases of optic neuritis developing after vaccinations for tuberculosis, hepatitis B, rabies, tetanus, meningitis, anthrax, mumps, measles, and rubella [1]. The influenza vaccine is also associated with a higher incidence of optic neuritis [1]. Optic neuritis following MMR vaccination usually develops between one week and a maximum of one month after administration of the vaccine [2–6]. However, a case of a patient who experienced a worsening vision as soon as only 24 hours after vaccination has been recorded [7].

## 2. Case Presentation

A healthy 30-year-old woman complained of headache and fever two days after administration of the MMR vaccine. About five days later, the patient reported a unilateral blurring of vision. She underwent a full ophthalmologic examination at the emergency department of the Ophthalmology Clinic in Trieste General Hospital. Visual acuity was found to be 20/20 in both eyes and the slit-lamp examination showed an anterior segment without signs of inflammation. Intraocular pressure was 14 mmHg in both eyes. On fundus examination of the right eye the optic disc appeared to be normal whereas the left eye optic disc margin was blurred. Fluorescein Angiography (FA) of the left eye showed the edges of the optical disc to be blurred, revealing the early stages of hyperfluorescence. There was no other angiographic evidence of disease in the left eye. Moreover, the right eye was within the normal angiographic range. Two color photographs of the fundus were taken confirming the presence of optic nerve head edema.

**Figure 1:**
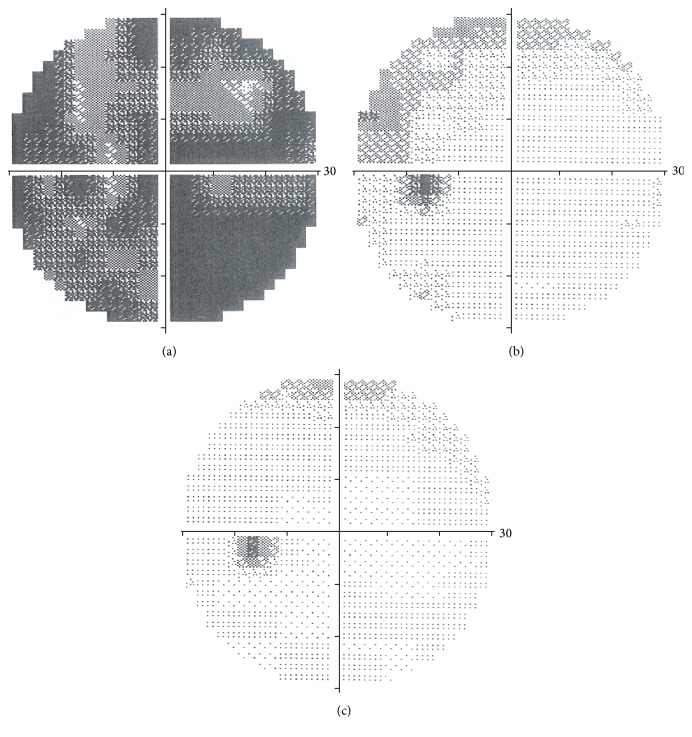
Visual field. (a) In acute phase. (b) After 5 days from treatment. (c) After 40 days from treatment.

On the third day after the first examination visual acuity was found to be 20/100 BCVA. The patient was treated with 500 mg i.v. of methylprednisolone for four days. The Computerized Perimetry Test (Figure 1(a)) revealed an overall reduction in the visual field. Electrophysiological exams were performed: the Visual Evoked Potentials (VEP) showed an increased latency and a reduction in amplitude (Table 1).

Additional radiological investigations were deemed necessary: a CT head scan, with and without contrast medium, and magnetic resonance imaging (MRI) of the brain, with and without contrast medium. Following administration of the contrast medium, the CT scan disclosed a nonhemorrhagic ultradense lesion with no signs of focal encephalitis injuries. The MRI revealed minor thickening of the left optic nerve, corresponding to an increased intensity of the T2 signal within a framework involving hyperemia and edema.

Four days of infusion therapy was followed by ten days of oral corticosteroid therapy, with a gradually diminishing dosage. Visual acuity remained stable at 20/100 BCVA for the next five days and was followed by a gradual improvement in visual acuity. The Computerized Perimetry Test was repeated (Figure 1(b)) and showed a significant improvement. A scotoma remained in a peripheral superonasal position in the left eye. After forty days, visual acuity was found to be 20/20 BCVA and the visual field was within normal limits (Figure 1(c)). Electrophysiological exams revealed an improvement, with a lower VEP latency and an increased amplitude (Table 2).

## 3. Discussion

The Measles-Mumps-Rubella vaccine consists of MMR and live attenuated virus prepared in baby chick embryo cell cultures.

Adverse events associated with the MMR vaccination can vary from simple local pain to rare systemic disorders, such as anaphylactic shock. Over the last thirty years, six cases of optic neuritis following vaccination against measles, mumps, and rubella have been reported.

Kazarian and Gager [2] reported the case of a 6-year-old boy who developed bilateral optic neuritis only 18 days after administration of the trivalent vaccine. Kline et al. [3] described a case of a 31-year-old woman who developed bilateral optic neuritis 11 days after a vaccination for rubella. Riikonen [4] diagnosed a unilateral optic neuritis and subsequent multiple sclerosis four weeks after rubella vaccination. Stevenson et al. [5] reported two cases, both involving 13-year-olds who developed optic neuritis 2-3 weeks after vaccination for measles and rubella, Arshi et al. [7] described a case of rapid onset optic neuritis in which the complication came out just in few hours in a 16-year-old boy, and Moradian and Ahmadieh [6] reported two cases of optic neuritis with onset less than 24 hours following measles-rubella vaccination.

To the best of our knowledge, there are no papers in the literature describing a case of unilateral optic neuritis following MMR vaccination in an adult without known systemic inflammatory or autoimmune disorders. Our patient developed optic neuritis about five days after being given a dose of MMR vaccine. As in the cases described in the literature above, neurological complications occurred within a week to one month of the administration of the MMR vaccine, long enough to increase the antibody titer and antigen-antibody complexes. Damage was due to immune complex disease, which caused vascular lesions and led to hyperemia and perivascular inflammation, with consequent rupture of the blood-brain barrier [4, 5]. An etiopathogenetic mechanism is consistent with the foregoing condition.

Optic neuritis is one of the rare complications associated with the mumps, measles, and rubella vaccine. This neurological disorder may be a toxic reaction to the nonviral component of the vaccine [4] and is probably caused by the antibody titer against live attenuated virus. It should be stressed that this is a temporary clinical situation, which resolves after administration of a high dose of corticosteroid in the initial phase of the therapy. However, it can lead to both unilateral and bilateral optic neuritis and can affect both sexes and all ages in subjects who undergo vaccination.

Another purpose of this case report is to underline the importance of assessing a patient's medical history and establishing the correlation between clinical symptoms and the MMR vaccine, not least because vaccination campaigns are recognized and supported by the WHO (World Health Organization) throughout the world.

## Figures and Tables

**Table 1:** Visual Evoked Potentials (VEP) in acute phase.

	VEP pattern 60′	VEP pattern 15′
Amplitude (uV)	2.93	2.42
Latency (ms)	109.57	109.57

**Table 2:** Visual Evoked Potentials (VEP) after 40 days from treatment.

	VEP pattern 60′	VEP pattern 15′
Amplitude (uV)	6.92	5.09
Latency (ms)	97.85	114.84

## References

[1] Stübgen J. P. (2013). A literature review on optic neuritis following vaccination against virus infections. *Autoimmunity Reviews*.

[2] Kazarian E. L., Gager W. E. (1978). Optic neuritis complicating measles, mumps, and rubella vaccination. *American Journal of Ophthalmology*.

[3] Kline L. B., Margulies S. L., Oh S. J. (1982). Optic neuritis and myelitis following rubella vaccination. *Archives of Neurology*.

[4] Riikonen R. (1989). The role of infection and vaccination in the genesis of optic neuritis and multiple sclerosis in children. *Acta Neurologica Scandinavica*.

[5] Stevenson V. L., Acheson J. F., Ball J., Plant G. T. (1996). Optic neuritis following measles/rubella vaccination in two 13-year-old children. *The British Journal of Ophthalmology*.

[6] Moradian S., Ahmadieh H. (2008). Early onset optic neuritis following measles-rubella vaccination. *Journal of Ophthalmic and Vision Research*.

[7] Arshi S., Sadeghi-Bazargani H., Ojaghi H. (2004). The first rapid onset optic neuritis after measles-rubella vaccination: case report. *Vaccine*.

